# Passamaquody Bay

**Published:** 1868-09

**Authors:** 


### PASSAMAQUODY BAY.

"ZYMOTIC."

There is an "Autocrat of the Breakfast Table" everywhere, at the country store and the two horse village, whose dictum is law and gospel to all the fawners and goslings in the neighborhood. On one occasion, down East, when a man was reading very fluently to a crowd at the post-office, he suddenly came to the phrase "Sine-Qua-Non," when he stopped, drew a long breath, and asked in great seriousness, "What's that?" "O! it's only an island in Passamaquody Bay, go on," said the village Johnson. We would like to inquire of the autocrat of the "Hub," what is "Zymotic"? There is a very intelligent gentleman in New York city who has been writing to the newspapers every week for a year or two, as a means of communicating to the people such information as is calculated to abate disease and render the city more healthful; we read those communications with considerable interest ; but it seems impossible for him to get through one of his epistles without the use of that big dictionary word. Many persons have a favorite word which they manage to lug in on all occasions. With some everything is "Prodigious ;" others think that "awful" is the most expressive word in the English language. "Horrid" has peculiar attractions with some of the gentler folk. Macauly speaks of one of the princes of the blood of England, whose whole stock in trade, in French at least, was "Ate-ill-posséeble," which he had managed to use to every one he spoke to, or who spoke to him. Any man of the least atom of common sense ought to know that the degraded classes of New York, among whom disease most abounds, are the most ignorant and most need information as to the means of taking care of themselves; they have no dictionaries to consult, or any acquaintances who would be likely to know the meaning of "Zymotic." Under date of August 1st, 1868, this "most learned judge" in matters pertaining to the public health, announces that in four weeks "1572 deaths were charged to ZYMOTIC diseases, and 185 to Insolation, or the immediate effects of excessive heat." How many in forty thousand of those most likely to suffer from this cause, know what "Insolation" is; or would know whether the writer meant that so many perished from one of two causes, or that "Insolation" and the immediate effects of excessive heat were one and the same thing; and if they were intended to be identical, how many would know that it simply meant "sun stroke," then why not use a plain word of two syllables, or two monosyllables, insted of seventeen and even these failing to give to the masses any intelligible idea?

As to the main word "Zymotic," we turn to the dictionary. Webster's latest and best says it "is any epidemic, endemic, contagious or sporadic affection which is produced by some morbific principle acting on the system like a ferment," which definition is just as clear as mud to the classes which die daily in the largest numbers of "Zymotic Diseases," and it is not at all likely that any one but a classical scholar would understand Webster's definition. The writer further says, that "among the poor and ignorant, a special kind of instruction and sanitary authority is needed." Well, let him begin right away and either instruct them as to the meaning of "Zymotic" or use some familiar English word, which will not require any definition, and which the "poor and ignorant" can comprehend without having to consult a ten dollar Dictionary and even then have no intelligible idea of the subject.

But since we are in search of a needle in two haystacks, to wit, Webster's English and Schrevelii's Greek dictionaries, let us not abandon the hunt for any trivial cause, but spend half a day in consulting the Astor Library. We shall not however go ourselves, but trust to the faithful memories of college days, in combination with the results of later scientific search; these lead us to the conclusion that Webster's definition is an absurdity, so far as the fundamental idea is concerned, that of "fermentation;" because that applies to dead matter, whereas, according to the present state of knowledge on the points in question, cholera, fever and ague, and kindred maladies are caused by living breathing things, hence "Zymotic" or Fermentive, in this connection, has no meaning. But there is an English word of two short syllables, of only five letters in all, which tells the whole story, and is perfectly intelligible to any child half a dozen years old; a word which is alike correct and applicable, whether the cause of these diseases be zoic or azoic, living or dead; that word is "dirty;" then it would read thus, that the great number of deaths stated as having occured in New York City, over eleven hundred in one week, were in large measure owing to the filthy living, or to be still more explicit, to dirty clothes, dirty skin, dirty houses, dirty yards, dirty kitchens, dirty food and dirty streets; then every person of two ideas could put them together and see and feel at once that he could and ought to do something towards saving the life and improving the health of himself, his family and of his neighborhood, but to talk for a year about "Zymotic" and all that nonsense, "get out." Hooray! for the dear old Anglo-Saxon, it beats all Greek, all Roman story, Zymotic, Dirty.

